# Plastome evolution and phylogeny of the tribe Ruteae (Rutaceae)

**DOI:** 10.1002/ece3.9821

**Published:** 2023-02-09

**Authors:** Qiaoyun Liu, Yongwei Gao, Wenpan Dong, Liangcheng Zhao

**Affiliations:** ^1^ School of Ecology and Nature Conservation Beijing Forestry University Beijing China; ^2^ Museum of Beijing Forestry University, Beijing Forestry University Beijing China

**Keywords:** chloroplast genome, evolution, phylogenetic analyses, Rutaceae, sequence divergence

## Abstract

Rutaceae is a large family, and the genus‐level classification in the subfamilies or tribes of this family is not unified based on different taxonomic treatments. Until now, phylogenetic relationships of some genera in traditional tribe Ruteae have not been clearly resolved. In this study, seven new complete plastomes of this tribe were sequenced, and a comparative analysis was performed to investigate their plastome characteristics and evolution. In addition, we inferred the phylogenetic relationships of Ruteae based on complete plastome and nuclear ITS data. All plastomes exhibited a typical quadripartite structure and were relatively conserved in their structure and gene arrangement. Their genome sizes ranged from 154,656 bp to 160,677 bp, and the size variation was found to be associated with differences in IR expansion and gene loss. A total of 112 to 114 genes were identified in the genomes, including 78 to 79 protein‐coding genes, 30 tRNA genes, 4 rRNA genes, and 2 pseudogenes. Sequence divergence analysis indicated that non‐coding regions exhibited a higher percentage of variable characters, and nine non‐coding and six coding regions were identified as divergent hotspots. Phylogenetic results based on different datasets showed that this tribe was divided into three reciprocally exclusive groups. The phylogenetic analyses between plastome and nuclear ITS data were partly incongruent with each other. This study provides new insights into plastome evolution of Ruteae as well as Rutaceae. The availability of these plastomes provides useful genomic resources for molecular DNA barcodes and phylogenetically informative markers and deepens our understanding of the phylogeny in Ruteae.

## Graphical abstract

Sequenced complete plastomes of Ruteae and analyze their genome characteristics and sequence variations. Identified divergence hotspots for useful molecular barcodes and phylogenetic markers. Provided new insights into the plastome evolution and phylogeny of Ruteae as well as Rutaceae.

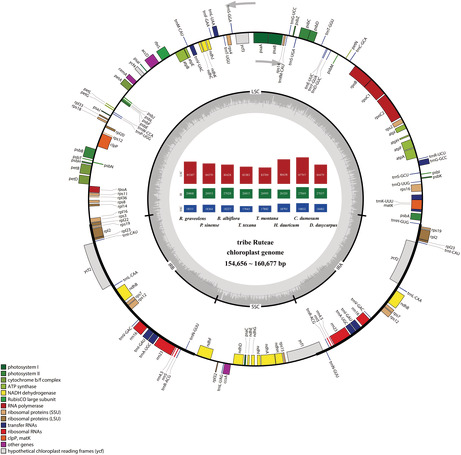

## 1 INTRODUCTION

Rutaceae is a large family (c. 154 genera and 2100 species) with great diversity in morphological characters and a nearly worldwide distribution (Appelhans et al., 2021; Groppo et al., 2022; Kubitzki et al., 2011; Zhang et al., 2008). This family comprises tall or short trees, shrubs or climbers, and rarely herbs. The relationships among the genera within the family are very complicated, and there have been considerably different taxonomic treatments (Appelhans et al., 2021; Chase et al., 1999; Groppo et al., 2008, 2012; Hartley, 1981; Kubitzki et al., 2011; Morton & Telmer, 2014; Poon et al., 2007; Scott et al., 2000; Waterman, 1975). The first comprehensive classification of the Rutaceae was undertaken by Engler (1896, 1931), in which 7 subfamilies, 12 tribes, and several subtribes were proposed. Among them, tribe Ruteae in the subfamily Rutoideae is characterized by an herbaceous or subshrub habit.

The Englerian traditional Ruteae has six genera: *Ruta* L. (including *Haplophyllum* A. Juss.), *Boenninghausenia* Rchb. ex Meisn., *Psilopeganum* Hemsl., *Thamnosma* Torr. & Frém., *Cneoridium* Hook. f., and *Dictamnus* L. Among them, *Ruta* includes seven to nine species of strongly scented herbs or half‐shrubs with a distribution from the Mediterranean region to southwest Asia (Kubitzki et al., 2011; Salvo et al., 2008). *Haplophyllum* was first proposed by Jussieu (1825) but placed in *Ruta* as a subgenus by Engler (1931). In later systematic works, the genus *Haplophyllum* was reinstated based on morphological and phytochemical features (Navarro et al., 2004; Townsend, 1986). It includes 66–68 species of herbs and half‐shrubs distributed in the western Mediterranean, northern Africa, Arabia, Central Asia, and China (Salvo et al., 2011). *Boenninghausenia* consists of one to three species of strongly scented herbs with a distribution from the Himalayas eastwards to Japan and southwards to Java and Lesser Sunda Islands (Kubitzki et al., 2011). Both *Psilopeganum* and *Cneoridium* are monotypic genera; the former is endemic to the narrow area of Central and Southwest China (Zhang et al., 2008), and the latter is restricted to southwestern North America. *Thamnosma* consists of eight shrubs or subshrubs species that occur in the southwestern United States, Somalia, and southern Africa. *Dictamnus* probably includes a single polymorphic species of strongly scented herbs widely distributed from warm‐temperate Europe through temperate Asia to North China (Kubitzki et al., 2011).

The traditional Ruteae was defined mainly by morphological characters, life forms, and habitat. However, generic taxonomy and relationship in this tribe are controversial (Gottlieb & Ehrendorfer, 1988; Kubitzki et al., 2011; Moore, 1936; Townsend, 1986). In the past decade or so, molecular sequence data have provided important insights into the intergeneric relationships of tribe Ruteae and related taxa. An early phylogenetic study using three plastid markers (Salvo et al., 2008) found that *Haplophyllum* and *Ruta* formed reciprocally exclusive monophyletic groups and that *Dictamnus* was not closely related to the other genera of Ruteae. A later study based on the same plastid markers and two nuclear markers was the first to include the genus *Psilopeganum* in the phylogenetic analysis of Ruteae (Appelhans et al., 2016). The results showed that *Psilopeganum* belonged to a group with *Boenninghausenia*, *Ruta*, and *Thamnosma*, but its exact position remained unclear. This study also showed that *Haplophyllum* and *Cneoridium* had a closer relationship with the subfamily Aurantioideae than with other genera of Ruteae. In the recent study on subfamily classification of Rutaceae, the genera of traditional Ruteae were placed into four subfamilies (Appelhans et al., 2021). *Boenninghausenia*, *Psilopeganum*, *Ruta*, *Thamnosma*, and another genus *Chloroxylon* DC. composed the subfamily Rutoideae. However, *Haplophyllum*, *Cneoridium*, and *Dictamnus* belonged to the subfamilies Haplophylloideae, Amyridoideae, and Zanthoxyloideae, respectively. Although different taxa of Ruteae and related genera were sampled in these phylogenetic studies, only a limited number of genetic markers were used to infer the phylogeny, and the resolution of some core nodes in the phylogenetic trees was low or lacking. Therefore, additional genetic characters are essential for resolving the phylogenetic relationships among the genera of Ruteae and related taxa.

Chloroplast genomes (plastomes) are usually circular DNA molecules composed of a large single copy (LSC) region and a small single copy (SSC) region separated by a pair of inverted repeat (IR) regions. Plastome is independent of nuclear genome and has highly conserved gene content, number, and structure (Barrett et al., 2014; Daniell et al., 2016). Over the past years, it has been used to elucidate plant molecular evolution, enhancing our understanding of chloroplast biology, intracellular gene transfer, conservation, and diversity (Daniell et al., 2016). Moreover, plastome sequences have been widely used to infer phylogeny relationships at different taxonomic levels and are sometimes used as DNA barcodes for species identification (Dong et al., 2012, 2021, 2022; Jansen et al., 2007). In recent years, the large number of complete plastome sequences has provided helpful data for modern taxonomy and phylogenetics and provided a higher resolution of evolutionary relationships within phylogenetic clades compared with small numbers of plastid or nuclear markers (Jansen & Ruhlman, 2012; Ma et al., 2014; Ruhfel et al., 2014; Daniell et al., 2016; Foster et al., 2018; Song et al., 2020).

To date, plastome characteristics and evolution in Ruteae at the generic level are not clear, and it is not available using the complete plastome data to infer the phylogeny of this tribe. In the present study, we first sequenced the complete plastomes of seven species in six genera (*Psilopeganum*, *Boenninghausenia*, *Thamnosma*, *Haplophyllum*, *Cneoridium*, and *Dictamnus*) and added the plastome of *Ruta graveolens* L. obtained from GenBank for comparative analyses. We attempted to reveal the plastome characteristics and evolution of the seven genera and provide useful plastid genomic resources for molecular DNA barcodes and phylogenetically informative markers. We then performed a phylogenomic analysis based on complete plastomes from 8 species of Ruteae, 15 other Rutaceae species, and 3 outgroup species from Simaroubaceae. In addition, we extended sampling and added the published plastid markers and nuclear internal transcribed spacer (ITS) sequences to construct the phylogenetic relationships of this tribe and related taxa to improve our understanding of the Ruteae phylogeny based on different data.

## 2 MATERIALS AND METHODS

### 2.1 Plant material, DNA extraction, and sequencing

Seven Rutaceae species were sampled and sequenced. The fresh leaves of *Boenninghausenia albiflora* (Hook.) Rchb. ex Meisn., *Dictamnus dasycarpus* Turcz., and *Haplophyllum dauricum* (L.) G. Don were collected from the field and dried with silica gel. Voucher specimens were deposited at the Museum of Beijing Forestry University, Beijing. The dry leaves of *Psilopeganum sinense* Hemsl. were sampled from the herbarium specimen in the Museum of Beijing Forestry University (BJFC). The total DNA of these four species was extracted from the dry leaves using the cetyltrimethylammonium bromide (CTAB; Doyle & Doyle, 1987) method. Total DNA of *Cneoridium dumosum* (Nutt.) Hook.f. ex Baill., *Thamnosma texana* (A. Gray) Torr., and *T. montana* Torr. & Frém was obtained from the DNA Bank of China, the Institute of Botany, Chinese Academy of Sciences (CAS). Details of the seven species sampling information are shown in Appendix A. The genome DNA was fragmented to construct 350 bp insert libraries. Paired‐end libraries were prepared with the DNA Library Preparation Kit (Illumina, San Diego, California, USA). Average 150 bp pair‐end reads were performed on an Illumina HiSeq X‐ten platform at Novogene Biotech Co. (http://www.novogene.com, China).

### 2.2 Genomes assembly and annotations

Complete plastome and ITS sequences of the seven species were assembled using GetOrganelle v1.6.2d with default parameters (Jin et al., 2020). The GetOrganelle first filtered plastid‐like reads, conducted the de novo assembly, purified the assembly, and finally, generated the complete plastid genomes (Bankevich et al., 2012; Camacho et al., 2009; Langmead & Salzberg, 2012). K‐mer gradients for a mean and maximum 150 bp reads were set as “‐k 21, 45, 65, 85,105” for all species. Bandage (Wick et al., 2015) was used to visualize the final assembly graphs to authenticate the automatically generated plastid genome. Gene annotation was conducted using Plann (Huang & Cronk, 2015) with the annotated plastome of *Ruta graveolens* (GenBank accession NC_045946) as the reference genome.

### 2.3 Characterization of repeat sequences

Repeat sequences were analyzed to calculate the content of dispersed and tandem repeats. Dispersed repeats were detected following Lee et al. (2020). Tandem Repeats Finder version 4.09 (Benson, 1999) was used to identify tandem repeats in the plastomes with default alignment parameters of match, mismatch, and insertions and deletions (indels) of 2, 7, and 7, respectively. The proportion of total repeats (length of repeats/length of plastome [IRa excluded]) was calculated for each plastome. Simple sequence repeats (SSRs) were detected using the PERL script MicroSAtellite (MISA) (Thiel et al., 2003) with a size motif of one to six nucleotides and a threshold of 10, 5, 4, 3, and 3 for mono‐, di‐, tri‐, tetra‐, penta‐, and hexa‐nucleotide, respectively.

### 2.4 Genome comparison and nucleotide divergence

The mVISTA program (http://genome.lbl.gov/vista/mvista/about.shtml) in the Shuffle‐LAGAN mode (Frazer et al., 2004) was used with the *R. graveolens* annotation as a reference to compare the plastomes. To screen for polymorphic hotspots, 77 shared protein‐coding genes (coding regions) and 107 intergenic spacer regions and introns (non‐coding regions) of the plastomes were extracted separately. All the regions were aligned using MAFFT (Katoh et al., 2019) and manually adjusted using MEGA 7 (Kumar et al., 2016). The aligned sequence length, number of variable sites, and number of parsimony informative characters (PICs) for each region were calculated using DnaSP 5.0 (Librado & Rozas, 2009). The variable sites include singleton and parsimony information sites. We used two methods to identify the mutation hotspots. The first was based on the percentage of variable sites, which calculated all the single‐nucleotide substitutions and included the most divergence markers. The second was based on the percentage of PICs, which included the most informative markers (Ma et al., 2018; Meiklejohn et al., 2016).

### 2.5 Phylogenetic analysis

To infer the phylogeny of Ruteae, three datasets were created. The first dataset CCG included 23 complete plastomes (7 newly generated and 16 obtained from GenBank) from 20 genera of Rutaceae, plus three outgroup sequences from Simaroubaceae (Appendix A and B). The second dataset CCG + PM combined CCG and the published sequences of plastid markers (*matK*, *rpl16*, and *trnL*‐*trnF*) from 27 species of traditional Ruteae (Appendix C), representing as many taxa and individuals of this tribe as possible. In addition, we also included the available *matK* and *trnL‐trnF* sequences of *Chloroxylon* in this dataset to test its phylogenetic relationship with regard to Ruteae (Appendix C). The third ITS dataset included 24 nuclear ITS sequences (7 newly created with the complete plastomes and 17 obtained from GenBank which are the same species but not the same samples as the plastomes) (Appendix B). All the sequences were aligned with MAFFT (Katoh et al., 2019), and ambiguous alignment regions were trimmed by Geneious 2021 with default parameters (Sites containing: Gaps = 20%) (Ripma et al., 2014).

The best‐fit model of nucleotide substitution was selected using the ModelFinder program (Kalyaanamoorthy et al., 2017) under the Bayesian information criterion. Maximum‐likelihood (ML) trees were inferred with the best GTR + F + R4 model from ModelFinder using the IQtree v1.6.12 (Nguyen et al., 2015), and 1000 bootstrap replicates were tested to evaluate the branch support values. The support values were marked in the strict tree (the consensus tree). Bayesian inference (BI) was executed with MrBayes v3.2.3 (Ronquist et al., 2012) in the GTR + G model, set to run for 2 million generations with four chains, sampled every 100 generations, with all other settings left at their defaults and 25% of the trees discarded as burn‐in. A maximum clade credibility tree was generated in TreeAnnotator using the rest trees. Phylogenetic consensus trees were visualized and edited using FigTree v.1.4.4 (Rambaut, 2018).

## 3 RESULTS

### 3.1 Features of plastomes

**FIGURE 1:**
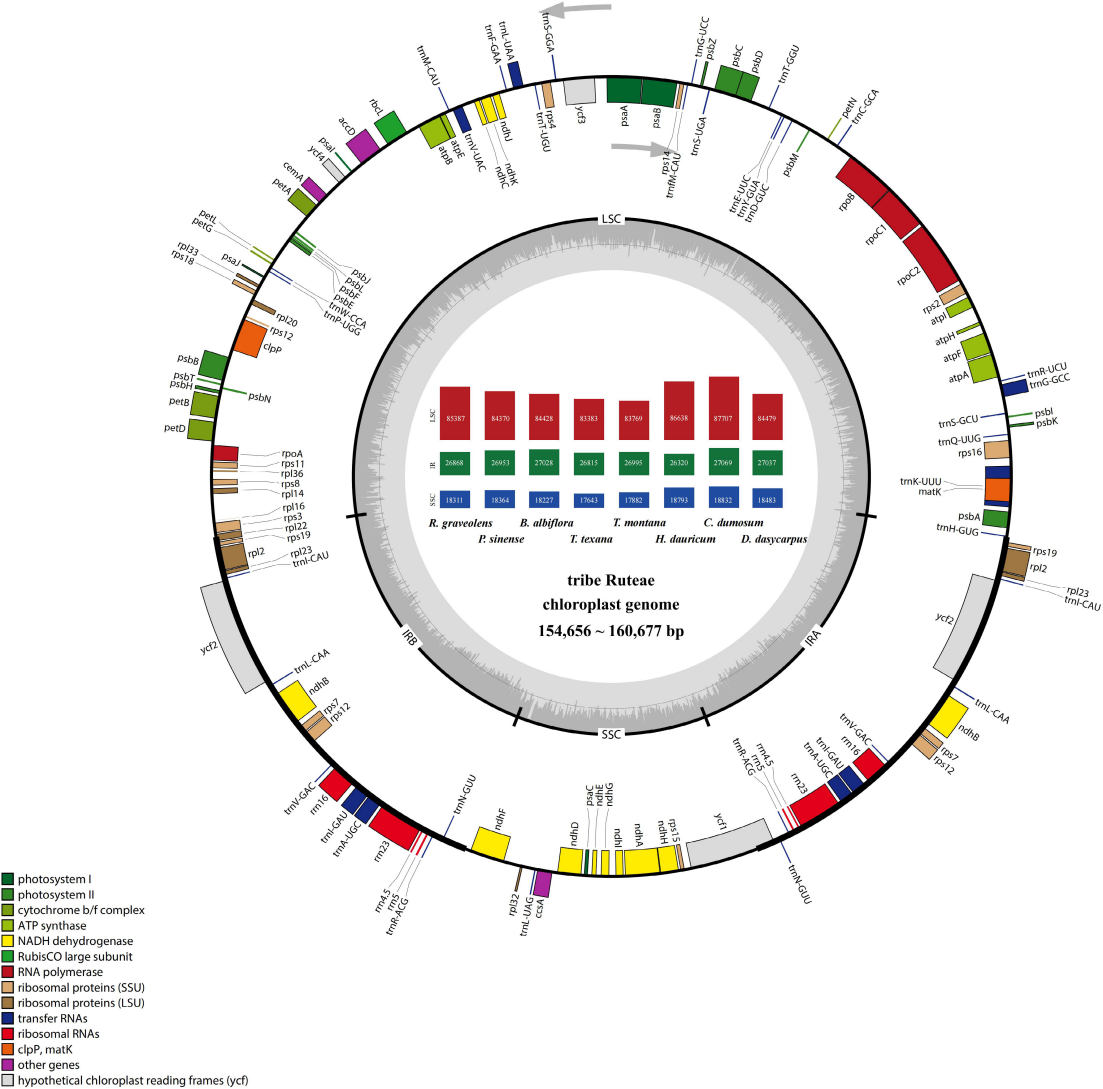
Gene map of *Psilopeganum sinense*. Genes shown inside the inner circle are transcribed counterclockwise and those outside are transcribed clockwise. Genes belonging to different functional groups are color coded. Darker gray in the inner circle corresponds to the GC content of the cp genome. The genome length information of seven newly sequenced plastomes was shown on the inside of the circle.

Seven newly assembled plastomes (*P. sinense*, *B. albiflora*, *H. dauricum*, *C. dumosum*, *T. texana*, *T. montana*, and *D. dasycarpus*) and one published plastome (*R. graveolens*) obtained from GenBank, representing seven genera, were used for comparative analyses. Each plastome from the eight species was a typical covalently closed, double‐stranded circular molecule that contained an LSC region of 83,383 bp–87,707 bp, an SSC region of 17,643 bp–18,832 bp, and a pair of IR regions of 26,320 bp–27,069 bp (Figure 1; Table 1). However, the eight species exhibited obvious variation in plastome size: the largest was *C. dumosum* (160,677 bp) and the smallest was *T. texana* (154,656 bp). The total GC content of the eight plastomes was 38.3%–39.1%. The GC contents of the LSC region, SSC region, and IR regions were 36.7%–37.7%, 32.8%–34.4%, and 42.9%–43.0%, respectively (Table 1).

**TABLE 1:** General features of the eight plastomes.

	*Ruta graveolens*	*Psilopeganum sinense*	*Boenninghausenia albiflora*	*Thamnosma texana*	*Thamnosma montana*	*Haplophyllum dauricum*	*Cneoridium dumosum*	*Dictamnus dasycarpus*
Genome size (bp)	157,434	157,000	156,711	154,656	155,641	158,017	160,677	157,036
LSC length (bp)	85,387	84,730	84,428	83,383	83,769	86,638	87,707	84,479
SSC length (bp)	18,311	18,364	18,227	17,643	17,882	18,739	18,832	18,483
IR length (bp)	26,868	26,953	27,028	26,815	26,995	26,320	27,069	27,037
Coding (bp)	90,712	90,896	91,092	90,810	91,028	90,764	91,296	91,100
Intron size (bp)	20,467	20,314	19,118	19,140	19,160	19,153	19,394	19,367
Spacer size (bp)	46,255	46,003	46,672	44,706	45,624	48,271	50,174	46,752
Number of genes	113	113	113	113	113	112	113	114
Number of protein‐coding genes	79	79	79	79	79	78	79	80
Number of tRNA genes	30	30	30	30	30	30	30	30
Number of rRNA genes	4	4	4	4	4	4	4	4
Total GC (%)	38.8	39	38.9	39.1	39.1	38.3	38.4	38.5
LSC of GC (%)	37.3	37.6	37.5	37.7	37.6	36.7	36.7	36.9
SSC of GC (%)	33.7	33.9	33.7	34.4	34.2	32.8	33.0	33.2
IR of GC (%)	42.9	42.9	42.9	42.9	42.9	43.0	42.9	42.9

There are 113 coding genes (including 79 protein‐coding genes) in the plastomes of *Ruta*, *Psilopeganum*, *Boenninghausenia*, *Thamnosma*, and *Cneoridium*, 112 genes (including 78 protein‐coding genes) in the plastome of *Haplophyllum*, and 114 genes (including 80 protein‐coding genes) in that of *Dictamnus*. Among these plastomes, *Haplophyllum* lost the *ycf15* gene from both the IRa and IRb regions, and the *infA* gene was missing from the LSC region in all genera except *Dictamnus*. All the plastomes had 30 tRNA genes and 4 rRNA genes. Seven protein‐coding genes (*rps19*, *rpl2*, *rpl23*, *ycf2*, *ycf15*, *ndhB*, and *rps7*) in five genera (except *Dictamnus* and *Haplophyllum*), seven tRNA genes (*trnI‐CAU*, *trnL‐CAA*, *trnV‐GAC*, *trnI‐GAU*, *trnA‐UGC*, *trnA‐UGC*, and *trnR‐ACG*) and four rRNA genes (*rrn16*, *rrn23*, *rrn4.5*, and *rrn5*) in all genera were duplicated in the IR regions (Figure 1; Table 1).

### 3.2 Repeats and SSR analysis

Repeated sequences have played key roles in plastome rearrangement, divergence, and evolution, and SSRs have been used extensively in population genetics and molecular identification (Guisinger et al., 2010). Repeat analyses revealed that the repetitive DNA has minor variation among the eight plastomes (Table 2). The highest proportion of dispersed repeats were identified in *H. dauricum* (1360 bp, 1.1%), followed by *C. dumosum* (1216 bp, 0.9%). They were slightly higher than the other six species (Table 2). The greatest amount and proportion of tandem repeats were identified also in *H. dauricum* (3657 bp, 2.8%), followed by *C. dumosum* (1867 bp, 1.4%). They were greater than *R. graveolens* (1490 bp, 1.1%) and *B. albiflora* (1356 bp, 1.1%). *P. sinense*, *D. dasycarpus*, *T. texana*, and *T. montana* had fewer tandem repeats (Table 2).

**TABLE 2:** Statistics of dispersed and tandem repeats in the eight plastomes.

	*Ruta graveolens*	*Psilopeganum sinense*	*Boenninghausenia albiflora*	*Thamnosma texana*	*Thamnosma montana*	*Haplophyllum dauricum*	*Cneoridium dumosum*	*Dictamnus dasycarpus*
Dispersed repeats (DRs)								
Length of DR	1024	1104	1056	976	864	1360	1216	976
GC (%) of DR	30.5	31.9	36.2	32.1	35.9	24	28.6	30.7
GC (%) without DR	38.1	38.3	37.8	38.3	38.3	37.5	37.6	37.6
Percentage of DR in Genome	0.8	0.8	0.8	0.8	0.7	1.1	0.9	0.7
Tandem repeats (TRs)								
Length of TR	1490	943	1356	795	689	3657	1867	889
GC (%) of TR	19.4	21.7	24	21.9	26	11.2	21	24.2
GC (%) without TR	38.2	38.3	38.2	38.4	38.4	38.1	37.7	37.7
Percentage of TR in genome	1.1	0.7	1.1	0.6	0.5	2.8	1.4	0.7
Total repeats								
Length of total repeats	2514	2047	2412	1771	1553	5017	3083	1865
GC (%) of total repeats	23.9	27.2	29.3	27.5	31.5	14.7	24.0	27.6
GC (%) without total repeats	38.3	38.4	38.3	38.4	38.4	38.3	37.8	37.7
Percentage of total repeats in genome	1.9	1.6	1.9	1.4	1.2	3.8	2.3	1.4

**FIGURE 2:**
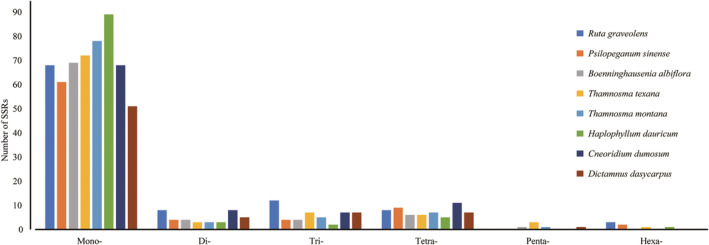
Classification of SSRs in the eight plastomes by repeat type. mono‐: mononucleotides; di‐: dinucleotides; tri‐: trinucleotides; tetra‐: tetranucleotides; penta‐: pentanucleotides; and hexa‐: hexanucleotides.

The eight plastomes were scanned for six types of SSRs (mono‐, di‐, tri‐, tetra‐, penta‐, and hexanucleotide) using the microsatellite identification tool MISA (Figure 2; Table 3). Mononucleotide‐to‐tetranucleotide SSRs were detected in all eight species, and mononucleotides were the predominant type. Pentanucleotide SSRs were found only in *B. albiflora*, *T. texana*, *T. montana*, and *D. dasycarpus*, and hexanucleotide SSRs were found only in *R. graveolens*, *P. sinense*, *T. texana*, and *H. dauricum*. It is noteworthy that no penta‐ and hexanucleotides were found in *C. dumosum*. In total, we identified 99, 80, 84, 91, 94, 100, 94, and 71 SSRs in the plastomes of *R. graveolens*, *P. sinense*, *B. albiflora*, *T. texana*, *T. montana*, *H. dauricum*, *C. dumosum*, and *D. dasycarpus*, respectively, and the mononucleotide repeat unit (A/T) was the most abundant in all SSRs (Table 3).

**TABLE 3:** Types and amounts of SSRs in the eight plastomes.

SSR type	Repeat unit	*Ruta graveolens*	*Psilopeganum sinense*	*Boenninghausenia albiflora*	*Thamnosma texana*	*Thamnosma montana*	*Haplophyllum dauricum*	*Cneoridium dumosum*	*Dictamnus dasycarpus*
Mono	A/T	68	61	67	68	77	89	68	50
	C/G	—	—	2	4	1	—	—	1
Di	AT/AT	8	4	4	3	3	3	8	5
Tri	AAG/CTT	5	4	3	5	5	—	3	1
	AAT/ATT	7	—	1	2	—	2	4	6
Tetra	AAAG/CTTT	3	2	1	2	2	1	4	2
	AAAT/ATTT	4	5	3	2	2	2	5	2
	AATG/ATTC	—	1	1	1	2	—	—	1
	ACAT/ATGT	—	—	—	—	—	—	—	1
	AGAT/ATCT	1	1	1	1	1	1	—	1
	AAGT/ACTT	—	—	—	—	—	1	—	—
	AACT/AGTT	—	—	—	—	—	—	1	—
	AATG/ATTC	—	—	—	—	—	—	1	—
Penta	ACACG/CGTGT	—	—	1	—	—	—	—	—
	AATAT/ATATT	—	—	—	—	1	—	—	—
	AAAAT/ATTTT	—	—	—	1	—	—	—	—
	AAATG/ATTTC	—	—	—	1	—	—	—	—
	AATAG/ATTCT	—	—	—	—	—	—	—	1
Hexa	AAATTC/AATTTG	—	—	—	—	—	1	—	—
	AATATG/ATATTC	—	2	—	—	—	—	—	—
	AATTAG/AATTCT	2	—	—	—	—	—	—	—
	AATTCG/AATTCG	1	—	—	—	—	—	—	—
	AATGGG/ATTCCC	—	—	—	1	—	—	—	—

### 3.3 IR contraction and expansion

**FIGURE 3:**
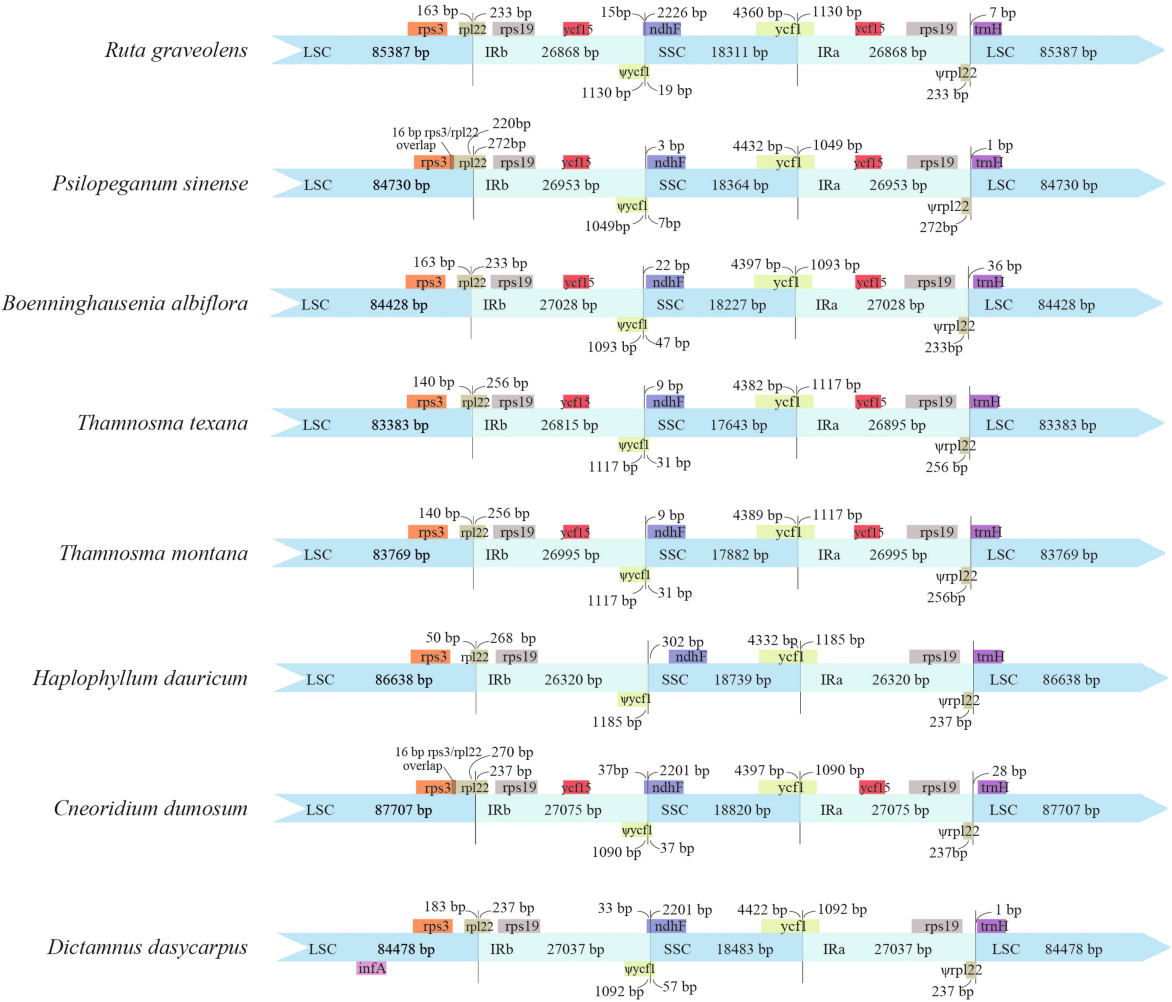
Comparison of the borders of LSC, SSC, and IR regions among the eight plastomes. Different color‐specific genes.

The IR regions of the eight plastomes ranged in length from 26,320 bp (*H. dauricum*) to 27,069 bp (*C. dumosum*), and the genes *rps3*, *rpl22*, *rps19*, *ndhF*, *ycf1*, and *trnH* were located at the junctions of the LSC/IR and SSC/IR borders (Figure 3). In the LSC/IRb border, the *rps19* gene in the LSC region was expanded completely to the IRb region in all species. The junctions were all crossed by the *rpl22* gene with a length from 318 bp to 492 bp, and the IRb expansion in *P. sinense* and *C. dumosum* resulted in a 16 bp overlap between *rpl22* and *rps3* in the LSC region. On the other hand, a 233–272 bp truncated *rpl22* pseudogene was located in the IRa/LSC border. As a result, the *rpl22* genes in all these species lost their protein‐coding ability because they were partially duplicated in the IRb region. In the IRb/SSC border, the *ndhF* gene crossed the junction and extended into the IRb region in *R. graveolens* (15 bp), *C. dumosum* (37 bp), and *D. dasycarpu* (33 bp). In the SSC/IRa borders, the junctions were all crossed by the *ycf1* gene with a length from 1030 bp to 1185 bp. Similarly, *ycf1* was partially duplicated in the IRb region. In the IRa/LSC borders, all the junctions were within the region between *rps19* and *trnH*.

### 3.4 Plastome sequence divergence

**FIGURE 4:**
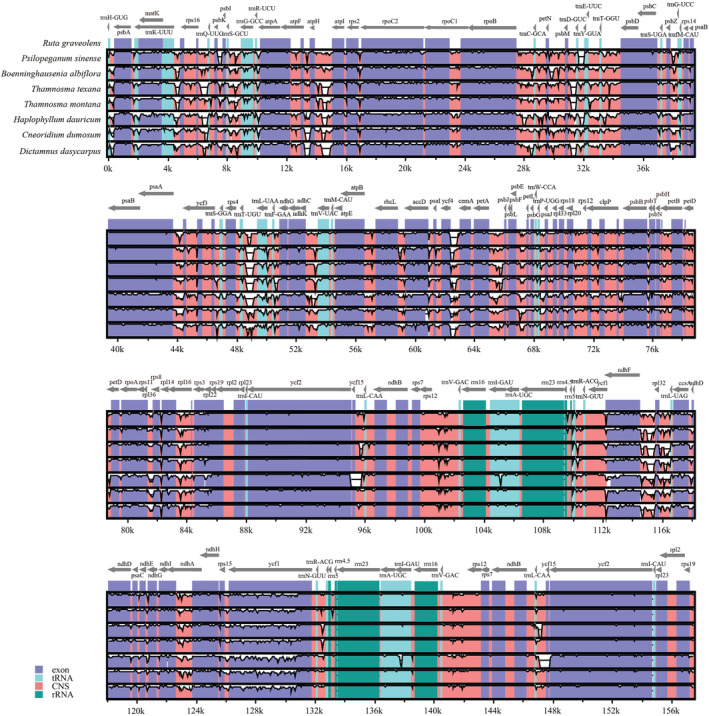
Sequence alignment of the eight plastomes using mVISTA under the LAGAN model.

To characterize genome divergence, multiple sequence alignments were performed between the eight plastomes using mVISTA. The comparison demonstrated that these plastomes had high consistency in gene arrangement (Figure 4). The eight plastomes dataset had an aligned length of 169,666 bp, with 16,354 variable sites (9.64%) and 6263 parsimony informative characters (PICs, 3.69%) (Table 4). The distribution pattern of variable sites varied greatly between coding and non‐coding regions and among LSC, SSC, and IR regions. The average variable percentages of coding regions and non‐coding regions were 8.18% and 10.82%, respectively. The SSC region exhibited the highest variation (17.80%), followed by the LSC region (11.93%), and the IR region (only 2.50%) (Table 4). The PICs percentages of coding regions and non‐coding regions were 3.12% and 4.15%, respectively. The SSC region had the highest PICs percentage (6.93%), followed by the LSC region (4.59%), and the IR region (only 0.88%) (Table 4).

**TABLE 4:** Statistics for genomic divergence among the eight plastomes.

Region	Aligned length of sequences (bp)	Variable sites		Parsimony informative characters (PICs)	
		Number	Variable percentage (%)	Number	PICs percentage (%)
SSC	20,149	3587	17.80	1397	6.93
LSC	94,566	11,285	11.93	4339	4.59
IRs	27,534	688	2.50	242	0.88
All noncoding	79,213	8574	10.82	3285	4.15
All coding	92,322	7555	8.18	2881	3.12
Whole plastomes	169,666	16,354	9.64	6263	3.69

**FIGURE 5:**
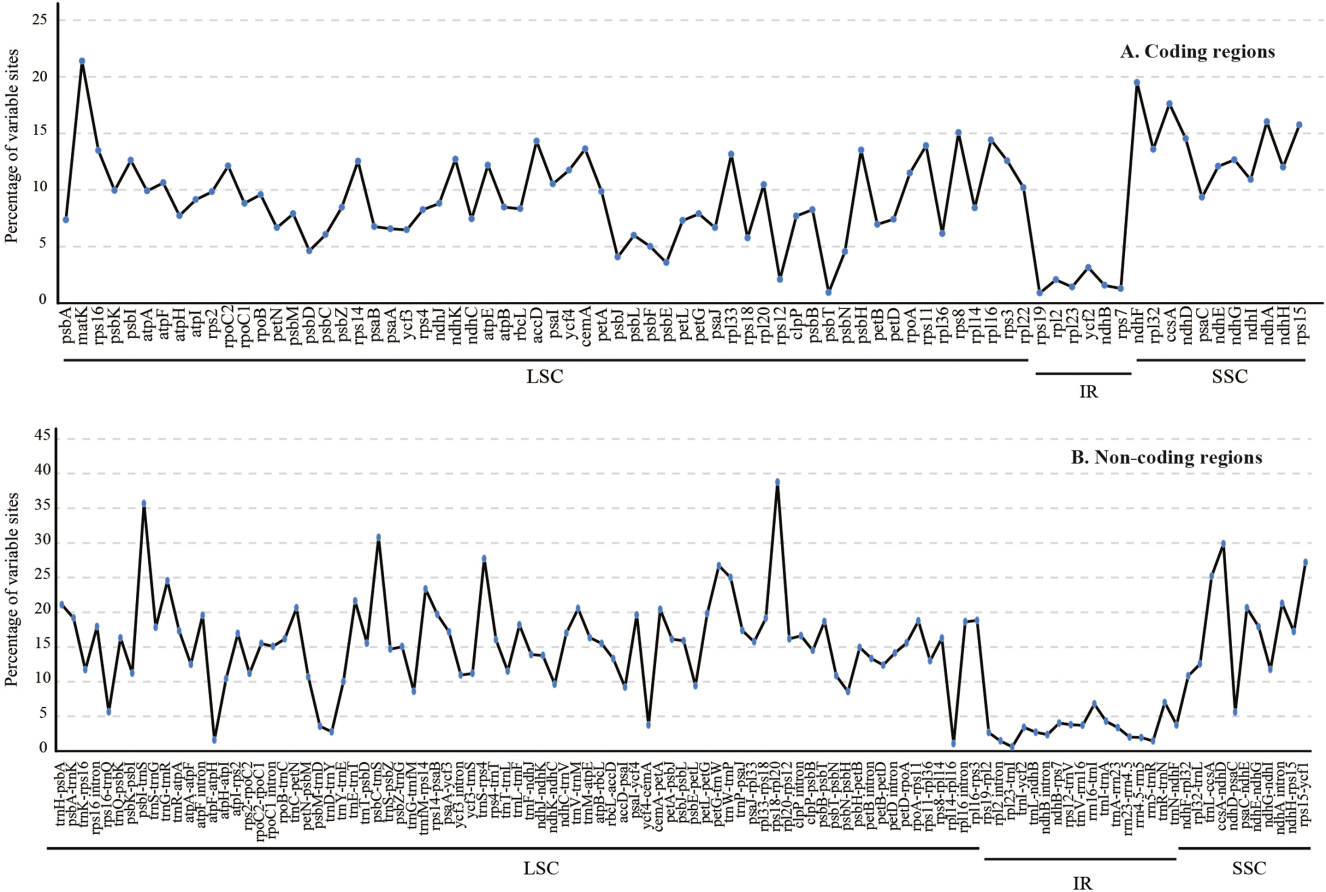
Percentage of variable sites in aligned sequences of the eight plastomes. A: coding region; b: non‐coding region.

To identify the mutation hotspot regions, the percentages of variable sites and PICs were calculated in each coding and non‐coding region (intergenic spacer regions and introns) (Figure 5). We defined a mutation hotspot region as having a percentage of variable sites >25% for non‐coding regions and > 15% for coding regions. Nine non‐coding regions (*rps18‐rpl20*, *psbI‐trnS*, *psbC‐trnS*, *ccsA‐ndhD*, *trnS‐rps4*, *rps15‐ycf1*, *petG‐trnW*, *trnL‐ccsA*, and *trnW‐trnP*) and six coding regions (*matK*, *ndhF*, *ccsA*, *ndhA*, *rps15*, and *rps8*) were identified as the most divergent hotspot regions (Table 5). Among them, *rps18‐rpl20*, *psbI‐trnS*, *psbC‐trnS*, *trnS‐rps4*, *petG‐trnW*, *trnW‐trnP*, *matK*, and *rps8* were located in the LSC region, whereas *ccsA‐ndhD*, *rps15‐ycf1*, *trnL‐ccsA*, *ndhF*, *ccsA*, *ndhA*, and *rps15* were located in the SSC region (Figure 1).

**TABLE 5:** Statistics for the most divergent hotspot regions identified in this study and the markers used in previous phylogenetic analyses (marked with asterisk).

Gene regions	Type	Aligned length of sequences (bp)	Variable sites		Parsimony informative characters (PICs)	
			Number	Variable percentage (%)	Number	PICs percentage (%)
*rps18‐rpl20*	Non‐coding	160	62	38.75	20	12.50
*psbI‐trnS (GCU)*	Non‐coding	143	51	35.66	16	11.19
*psbC‐trnS (UGA)*	Non‐coding	263	81	30.80	36	13.69
*ccsA‐ndhD*	Non‐coding	345	103	29.86	43	12.46
*trnS (GGA)‐rps4*	Non‐coding	321	89	27.73	37	11.53
*rps15‐ycf1*	Non‐coding	423	115	27.19	37	8.75
*petG‐trnW (CCA)*	Non‐coding	161	43	26.71	14	8.70
*trnL (UAG)‐ccsA*	Non‐coding	123	31	25.20	9	7.32
*trnW (CCA)‐ trnP (UGG)*	Non‐coding	188	47	25.00	22	11.70
matK*	Coding	1581	338	21.38	123	7.78
*ndhF*	Coding	2247	438	19.49	187	8.32
*ccsA*	Coding	954	168	17.61	66	6.92
*ndhA*	Coding	936	150	16.03	50	5.34
*rps15*	Coding	273	43	15.75	14	5.13
*rps8*	Coding	405	61	15.06	19	4.69
rpl16 intron*	Non‐coding	1160	216	18.62	78	6.72
trnL (UAA)‐trnF (GAA)*	Non‐coding	479	87	18.16	38	7.93
rps16 intron*	Non‐coding	977	175	17.91	73	7.47
atpB‐rbcL*	Non‐coding	890	138	15.51	46	5.17
rpl16*	Coding	444	64	14.41	33	7.43
atpB*	Coding	1497	127	8.48	61	4.07
rbcL*	Coding	1428	119	8.33	43	3.01

With regard to PICs, we defined the mutation hotspot regions as having a percentage > 11% for non‐coding regions and > 6% for coding regions. In non‐coding sequences, gene regions with a high percentage of PICs included *psbC‐trnS* (13.69%), *rps18‐rpl20* (12.50%), *ccsA‐ndhD* (12.46%), *trnW‐trnP* (11.70%), *trnS‐rps4* (11.53%), and *psbI‐trnS* (11.19%), and regions with lower percentages were *rps15‐ycf1* (8.75%), *petG‐trnW* (8.70%), *trnL (UAA)‐trnF (GAA)* (7.93%), *rps16* intron (7.47%), *trnL‐ccsA* (7.32%), *rpl16* intron (6.72%), and *atpB‐rbcL* (5.17%) (Table 5). Among the coding sequences, *ndhF*, *matK*, *rpl16*, and *ccsA* had higher percentages of PICs (8.32%, 7.78%, 7.43%, and 6.92%, respectively), whereas *ndhA*, *rps15*, *rps8*, *atpB*, and *rbcL* had lower percentages (5.34%, 5.13%, 4.69%, 4.07%, and 3.01%, respectively) (Table 5).

### 3.5 Phylogenetic analysis

**FIGURE 6:**
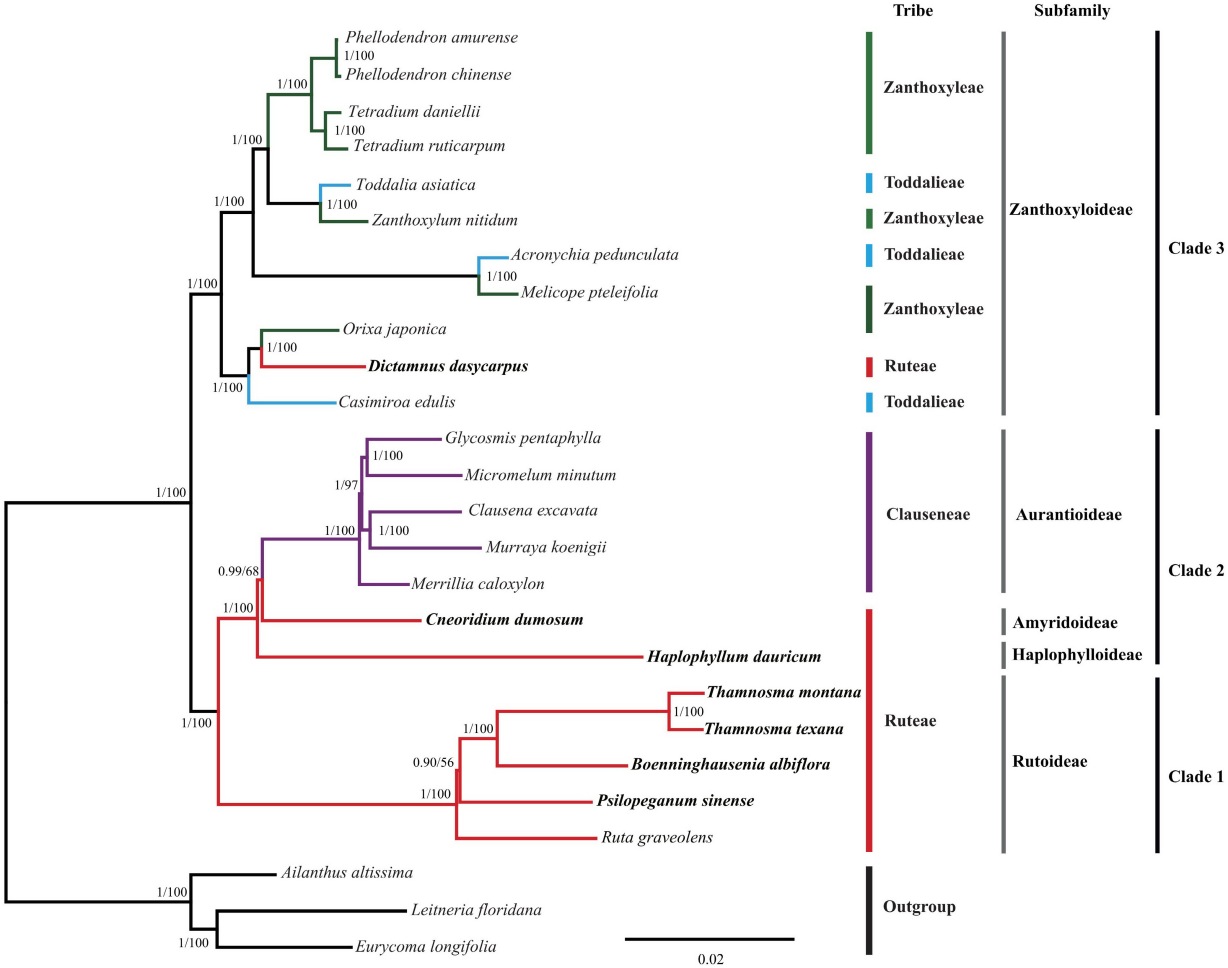
Phylogenetic reconstruction of Rutaceae and outgroup species using Bayesian inference (BI) and maximum‐likelihood (ML) methods based on CCG dataset. The support values for the nodes are for posterior probabilities (PP) and bootstrap support (BS). Tribes (bars) are based on Engler (1931) and subfamilies (bars) are based on Appelhans et al. (2021). The newly sampled species’ name was marked in bold.

**FIGURE 7:**
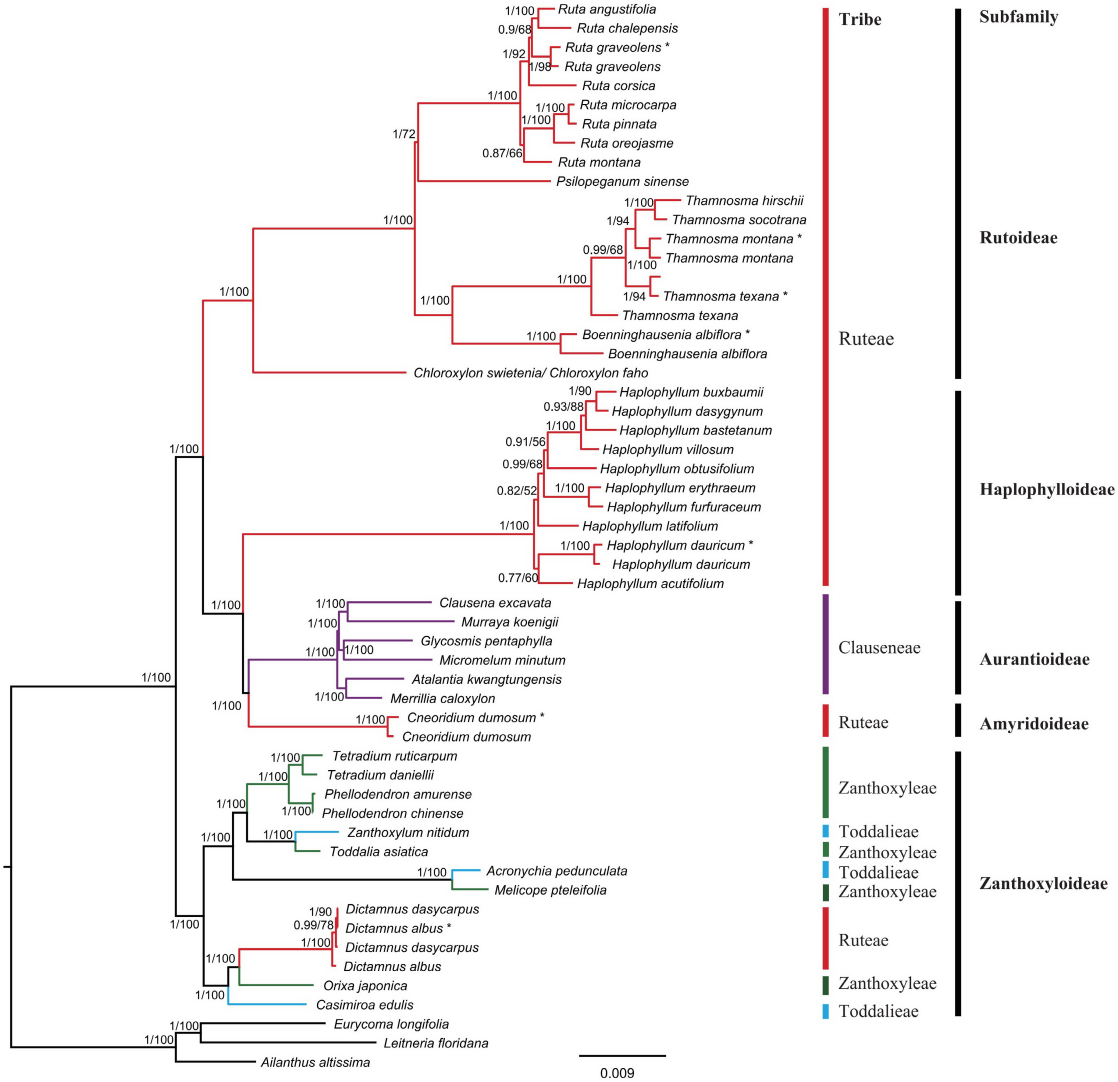
Phylogenetic reconstruction of Rutaceae and outgroup species using Bayesian inference (BI) and maximum‐likelihood (ML) methods based on CCG and CCG + PM datasets. The support values for the nodes are for posterior probabilities (PP) and bootstrap support (BS). Tribes (bars) are based on Engler (1931) and subfamilies (bars) are based on Appelhans et al. (2021). The species using complete plastomes were marked with asterisk.

BI and ML analyses based on CCG dataset and CCG + PM dataset indicated similar tree topology (Figures 6 and 7). The results showed that the traditional tribe Ruteae was polyphyletic and was split across three clades (Clade 1, Clade 2, and Clade 3). Clade 1 was composed of *Ruta*, *Psilopeganum*, *Boenninghausenia*, and *Thamnosma* and formed a highly supported monophyletic group that represented the core Ruteae. Within these four genera, *Boenninghausenia* and *Thamnosma* were sister genera with high support (PP/BS:1/100). For CCG dataset, *Psilopeganum* was sister to the *Boenninghausenia–Thamnosma* clade with weak support (PP/BS: 0.89/56); then, *Ruta* was sister to the *Psilopeganum* and *Boenninghausenia–Thamnosma* clade with high support (PP/BS:1/100). For CCG + PM dataset, however, *Ruta* was resolved as sister to *Psilopeganum* (PP/BS: 1/72). The genus *Chloroxylon* was sister to the core Ruteae with high support (PP/BS:1/100).

Clade 2 was composed of *Haplophyllum*, *Cneoridium*, and five genera of the tribe Clauseneae (subfamily Aurantioideae). These five genera were embedded in Clade 2, which formed a sister group with *Cneoridium*, then in turn with *Haplophyllum*. Clade 3 was composed of *Dictamnus*, *Casimiroa* La Llave, and *Orixa* Thunb. These three genera formed a highly supported monophyletic group (PP/BS: 1/100) closely related to the members of subfamily Zanthoxyloideae. *Orixa* and *Dictamnus* were resolved as highly supported sister genera (PP/BS: 1/100); they formed a monophyletic group that was in turn sister to *Casimiroa* with high support (PP/BS: 1/100).

**FIGURE 8:**
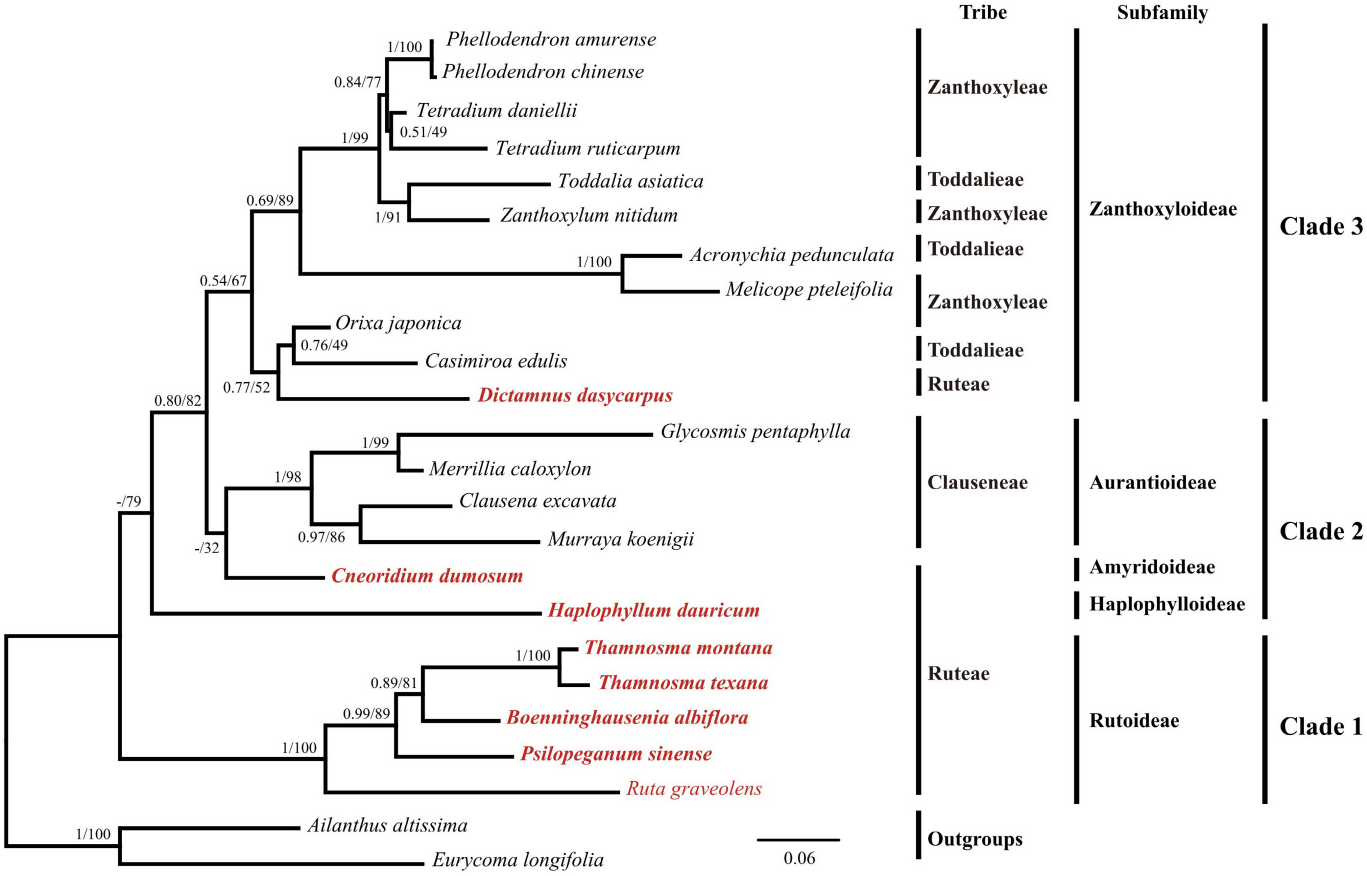
Phylogenetic reconstruction of Ruteae using Bayesian inference (BI) and maximum‐likelihood (ML) methods based on ITS dataset. The support values for the nodes are for posterior probabilities (PP) and bootstrap support (BS). Tribes and subfamilies (bars) are same as in Figure 6. The newly sampled species’ name was marked in bold.

The phylogenetic tree based on nuclear ITS dataset (Figure 8) showed that the four genera of *Ruta*, *Psilopeganum*, *Boenninghausenia*, and *Thamnosma* formed a monophyletic group with high support, and their relationships were consistent with the plastome tree. The significant incongruence between the two datasets involved the relationships of *Haplophyllum* and *Cneoridium*. In the ITS tree, *Haplophyllum* was sister to the clade of *Cneoridium*—Aurantioideae and Zanthoxyloideae—, which was very different from the plastome tree. In addition, in the ITS tree, *Dictamnus* was sister to *Orixa*–*Casimiroa*, but in the plastome tree, *Casimiroa* was sister to *Dictamnus*–*Orixa*. However, the support values in these topological incongruence nodes were very low.

## 4 DISCUSSION

### 4.1 Plastomes characteristics

We sequenced seven plastomes and compared eight plastomes representing all genera of the traditional tribe Ruteae in order to elucidate their plastome evolution. Their genome structures and gene orders were fairly conserved. All the genomes had a typical quadripartite structure composed of one LSC, one SSC, and two IR regions, and there were no rearrangements in genome organization (Figures 1 and 4). However, there was variation in gene content and gene number. GC content is an important genomic and systematic character that describes the nucleotide proportions in the genome. When GC content in the genome is greater, it has a greater density of DNA bases, making the sequence more stable and difficult to mutate (Sun et al., 2021). The overall GC content of the eight plastomes varied from 38.3% to 39.1% (Table 1) and was clearly lower than the AT content. The IR regions showed higher GC contents due to the abundance of rRNA and tRNA (Li et al., 2016).

Gene loss often occurs during the evolution of angiosperms plastomes (Feng et al., 2020; Lei et al., 2016). Among the eight plastomes examined here, the *ycf15* gene was lost in *H. dauricum* and *D. dasycarpu*, and the *infA* gene was missing in seven species (except *D. dasycarpu*) (Figure 3), indicating that gene deletion has occurred during the process of evolution. The *ycf15* gene, a hypothetical chloroplast open reading frame, is conserved among plants, although its function is unclear. In recent years, this gene has served as a useful DNA marker for plant species identification (Gao, 2017). The *infA* gene encodes the translation initiation factor 1 and is regarded as one of the most mobile genes, as it has been transferred many times between the cp and nuclear genomes in plants (Millen et al., 2001). Using the clean data, we mapped the reads to the sequence of *infA* gene. We found a homologous copy in *P. silopeganum* and the sequence had high divergence with the plastome copy, indicating that this gene has transferred to the nuclear or mitochondrial genome. However, this copy was not found in other species, indicating that *infA* may be completely lost.

The contraction and expansion of IRs generally lead to size variation in the plastomes of angiosperms and play important roles in genome evolution (Tanvi et al., 2017). Reduction of plastome size is caused mainly by IR contraction, gene loss, intron loss, or intergenic spacer loss (Dugas et al., 2015; Jansen et al., 2008). By contrast, increases in plastome size are usually caused by IR expansion and gene duplication (Dugas et al., 2015; Wang et al., 2018). The sizes of the eight plastomes varied from 154,656 to 160,677 bp. In all genomes, large IR expansions caused the *rps19* gene to completely change position from the LSC to the IRb region, and gene replication occurred in the IRa region (Figure 3). IR expansion also led to the partial duplication of *rpl22* and *ycf1* in the boundary region, thereby producing two pseudogenes (Figure 3).

### 4.2 Molecular markers

Complete plastome sequences can provide vast sequence information for identifying different taxa and inferring their phylogenetic relationships (Daniell et al., 2016; Dong et al., 2012). Here, most sequence variation occurred in the non‐coding sequences of the eight plastomes (Figure 5; Table 4), indicating that the coding regions were more conserved than the non‐coding ones, consistent with most angiosperm plastomes (Daniell et al., 2016; Feng et al., 2020). In particular, the IR regions were much less divergent than the LSC and SSC regions (Table 4). We identified nine non‐coding and six coding regions with the highest percentage of variation as divergence hotspots (Table 5). These divergent regions may be undergoing rapid nucleotide substitution, indicating great potential molecular markers for phylogeny and evolutionary research in Ruteae as well as Rutaceae, especially at the generic level.

Previous phylogenetic studies of Ruteae and related genera have used coding genes (*atpB*, *rbcL*, *rpl16*, and *matK*) and non‐coding markers (*atpB‐rbcL*, *trnL‐trnF*, *rpl16* intron, and *rps16* intron) (Appelhans et al., 2016, 2021; Chase et al., 1999; Groppo et al., 2008; Morton & Telmer, 2014; Salvo et al., 2008). We compared the sequence divergence between these markers and the current mutation hotspot regions. The percentages of variable sites and PICs in these markers are listed in Table 5. The results show that the four non‐coding markers and two coding markers (*atpB* and *rbcL*) are much less variable than the mutation hotspot regions identified in this study, and other two coding markers, *matK* and *rpl16*, have higher percentages of variable sites and PICs.

### 4.3 Phylogenetic analysis

We conducted phylogenomic analysis of the traditional tribe Ruteae and related taxa based on CCG dataset and CCG + PM dataset. For Clade 1, our results from both datasets showed that the core Ruteae consisted of *Ruta*, *Psilopeganum*, *Boenninghausenia*, and *Thamnosma*. Within the core Ruteae, the CCG tree suggested that *Ruta* was the first divergent group and sister to the other three genera with high support. The results also indicated that *Psilopeganum* was sister to the *Boenninghausenia*–*Thamnosma* clade, although the resolution of the *Psilopeganum* position was relatively low. By contrast, the CCG + PM tree suggested that *Ruta* and *Psilopeganum* formed a sister group with relatively low support, which then was sister to the *Boenninghausenia*–*Thamnosma* clade. Appelhans et al. (2016, 2021) used several plastid markers and found that *Psilopeganum* was the first divergent group, but without statistical support. Appelhans et al. (2016) also proposed two possible topologies for the sister group relationship of *Psilopeganum* or *Ruta* with the *Boenninghausenia*–*Thamnosma* clade: one is (*Psilopeganum*, (*Ruta*, (*Boenninghausenia*, *Thamnosma*))), and the other is (*Ruta*, (*Psilopeganum*, (*Boenninghausenia*, *Thamnosma*))). Based on the floral development research in *R. graveolens* and *P. sinense*, Wei et al. (2012) suggested that bicarpellate *Psilopeganum* most likely evolved from a *Ruta*‐like tetracarpellate ancestor. Based on our plastome analysis and their anatomical features, the relationships among the four genera of the core Ruteae are more likely to be (*Ruta*, (*Psilopeganum*, (*Boenninghausenia*, *Thamnosma*))). Moreover, the phylogenetic tree has a short internode connected by long branches at the *Ruta* and *Psilopeganum* nodes, suggesting that this clade may have undergone rapid radiation.

*Chloroxylon* is a small genus of three tree species that has traditionally been placed in the Engler's subfamily Flindersioideae (Kubitzki et al., 2011). However, previous phylogenetic studies at the family level resolved this genus as a close relative of *Ruta* (Chase et al., 1999; Groppo et al., 2008; Morton & Telmer, 2014). In the recent phylogenetic study, *Chloroxylon*, together with *Boenninghausenia*, *Psilopeganum*, *Ruta*, and *Thamnosma*, formed the Rutoideae subfamily and *Chloroxylon* was at the base of this subfamily (Appelhans et al., 2021). In our CCG + PM analysis, *Chloroxylon* belonged to the Ruteae tribe and was at the base of this tribe, consistent with the results of Appelhans et al. (2021). Considering that *Chloroxylon* is distributed in Madagascar, India, and Sri Lanka, this relationship suggested that the shrubby/herbaceous Ruteae may have a woody origin and an austral link through *Chloroxylon*.

For Clade 2, our results from both datasets supported the separation of *Haplophyllum* from *Ruta*, consistent with morphological findings (Townsend, 1986) and previous molecular studies (Appelhans et al., 2016, 2021; Salvo et al., 2008). Moreover, the phylogenetic trees showed that *Cneoridium* was sister to the clade formed by the genera of subfamily Aurantioideae, and *Haplophyllum* was at the base of this clade with high support. Although *Cneoridium*, *Haplophyllum*, and Aurantioideae are closely related, their relationships have not previously been clearly resolved. Manafzadeh et al. (2014) used three plastid markers and found that *Cneoridium* was sister to Aurantioideae, and *Haplophyllum* was at the base of this clade; however, based on six nuclear and plastid markers, Appelhans et al. (2021) found that *Haplophyllum* was sister to Aurantioideae, and *Cneoridium* and *Amyris* were at the base of this clade. Our data support the findings of Manafzadeh et al. (2014). Aurantioideae are characterized by baccate fruits (berries or hesperidia) (Appelhans et al., 2021). *Cneoridium* also possesses a globose, fleshy, punctate, and thin‐skinned berry. By contrast, the fruit of *Haplophyllum* is a typical capsule, dehiscing from the inner side of the apex (Kubitzki et al., 2011). The fruit morphology supported a closer relationship of *Cneoridium* with Aurantioideae. Similarly, the internode branch length was very short so this clade may have undergone rapid radiation.

For Clade 3, *Dictamnus* was nested within *Orixa* and *Casimiroa*, confirming the exclusion of *Dictamnus* from the tribe Ruteae, as documented previously (Appelhans et al., 2016; Salvo et al., 2008). Several studies based on different plastid markers found that *Dictamnus* had a closer relationship with the genera *Skimmia* Thunb. and/or *Casimiroa* (Appelhans et al., 2021; Chase et al., 1999; Groppo et al., 2008; Morton & Telmer, 2014). Due to the incongruence between morphological characters and molecular analyses of these three genera, Kubitzki et al. (2011) regarded them as isolated and unplaced genera of the subfamily Rutoideae. Very little is known about the phylogenetic relationship of *Orixa*, a monotypic genus that occurs in mountain forests of southern and middle Japan, Korea, and China. Previous studies showed that *Orixa*, *Skimmia*, and *Dictamnus* formed a clade, and *Orixa* was sister to *Skimmia* (Appelhans et al., 2021; Salvo et al., 2008). *Orixa* and *Dictamnus* have very different leaf and flower morphologies, but they share basally connate follicles with a cartilaginous endocarp that discharges elastically with seeds at dehiscence. They also share black, subglobose, shiny seeds with thin and brittle seed coats. By contrast, both *Skimmia* and *Casimiroa* have fleshy drupaceous fruit (Kubitzki et al., 2011; Zhang et al., 2008). The fruit and seed characters provide morphological support for the closer relationship between *Dictamnus* and *Orixa* inferred from our data, although whether these are synapomorphies will require further investigation.

Phylogenetic results between plastome and ITS datasets were partly incongruent with each other. The ITS tree confirmed the monophyletic group of the core Ruteae and supported the relationships of (*Ruta*, (*Psilopeganum*, (*Boenninghausenia*, *Thamnosma*))). However, there were conflicts concerning the phylogenetic positions of *Haplophyllum* and *Dictamnus*. Extensive incongruent patterns have been found between cp and nuclear data, most possibly caused by hybridization/introgression, incomplete lineage sorting (ILS), rapid radiation, and reticulate evolution (Degnan & Rosenberg, 2009; Dong et al., 2022; Lin et al., 2019; Pelser et al., 2010). In addition, sampling biases, inadequate nucleotide sequence data, and horizontal gene transfer (HGT) also have important impacts on incongruence (Meng et al., 2021; Soltis & Kuzoff, 1995). In this study, it is not possible to determine the underlying causes of the observed incongruences at the generic level and further study is needed. It is worth noting that most of the support values in the ITS tree were low, which was probably due to the inadequate variations provided by a limited number of DNA loci. Therefore, our results highlighted the advantage of using complete plastomes with more informative sites in resolving phylogenetic relationships of tribe Ruteae.

## 5 CONCLUSIONS

This study investigated the characteristics and evolution of eight plastomes, representing all the genera of the traditional tribe Ruteae (Rutaceae). The results revealed that the genome structure and gene order were highly conserved among these genera. However, the genome size exhibited a greater difference, which was related to both IR expansion and gene loss. The sequence divergence and mutation hotspots of the plastomes were analyzed. Nine non‐coding and six coding regions had the highest divergence and may be considered useful molecular barcodes and phylogenetic markers. The phylogenetic analysis based on different datasets confirmed the core Ruteae and supported separating traditional Ruteae into three reciprocally exclusive groups. This study revealed new insights into the plastome evolution in Ruteae as well as in Rutaceae, and indicated that the cp genomic data with obviously more informative sites could provide a deeper understanding of the phylogeny within Ruteae and related taxa.

## AUTHOR CONTRIBUTIONS

**Qiaoyun Liu:** Conceptualization (equal); data curation (lead); formal analysis (lead); investigation (lead); methodology (lead); resources (equal); software (lead); validation (equal); writing – original draft (equal). **Yongwei Gao:** Data curation (equal); formal analysis (equal); investigation (equal); methodology (equal); software (equal). **Wenpan Dong:** Conceptualization (equal); data curation (equal); formal analysis (equal); methodology (equal); resources (equal); writing – review and editing (equal). **Liangcheng Zhao:** Conceptualization (lead); data curation (equal); formal analysis (equal); funding acquisition (lead); investigation (equal); methodology (equal); resources (lead); supervision (lead); validation (equal); writing – original draft (equal); writing – review and editing (lead).

## FUNDING INFORMATION

This research was supported by the Science and Technology Basic Resources Investigation Program of China “Survey and Germplasm Conservation of Plant Species with Extremely Small Populations in South‐west China” (Grant No. 2017FY100100).

## CONFLICT OF INTEREST

The authors declare that there is no conflict of interest.

## Data Availability

The seven new sequenced plastomes were submitted to the NCBI database (https://www.ncbi.nlm.nih.gov/) with GenBank accession numbers MZ569021 (*Boenninghausenia albiflora*), MZ569022 (*Cneoridium dumosum*), MZ569023 (*Dictamnus dasycarpus*), MZ569025 (*Haplophyllum dauricum*), MZ531889 (*Psilopeganum sinense*), MZ569024 (*Thamnosma montana*), and MZ569026 (*Thamnosma texana*), respectively.

## APPENDIX A Sample information of the seven newly sequenced plastomes.

**Table:**

Species	Sample locality	Voucher	GenBank accession
*Boenninghausenia albiflora*	Dayi, Sichuan	BJFUZLC06004	MZ569021
*Cneoridium dumosum*	Institute of Botany, Chinese Academy of Sciences, Beijing	ENC851151	MZ569022
*Dictamnus dasycarpus*	Taituoshan, Hebei	BJFUZLC12010	MZ569023
*Haplophyllum dauricum*	Yanchi, Ningxia	BJFUZLC12026	MZ569025
*Psilopeganum sinense*	Museum of Beijing Forestry University, Beijing	BJFC00083452	MZ531889
*Thamnosma montana*	Institute of Botany, Chinese Academy of Sciences, Beijing	ENC851150	MZ569024
*Thamnosma texana*	Institute of Botany, Chinese Academy of Sciences, Beijing	ENC851149	MZ569026

## APPENDIX B List of species and their GenBank accessions for plastome and ITS sequences obtained from GenBank in the study. Outgroup species were marked with asterisk.

**Table:**

Species	GenBank accession	
	Complete plastomes	ITS sequence
*Acronychia pedunculata*	MW542636	KJ158057
*Ailanthus altissima**	NC_037696	KM051450
*Casimiroa edulis*	NC_046844	DQ225795
*Clausena excavata*	KU949003	FJ434152
*Eurycoma longifolia**	MH751519	MN715379
*Glycosmis pentaphylla*	NC_032687	FJ434151
*Leitneria floridana**	NC_030482	—
*Melicope pteleifolia*	NC_053871	MG595162
*Merrillia caloxylon*	NC_032688	FJ434149
*Micromelum minutum*	NC_032689	—
*Murraya koenigii*	NC_032684	JX144213
*Orixa japonica*	MW574915	MH711814
*Phellodendron amurense*	NC_035551	MK419239
*Phellodendron chinense*	NC_050949	KT961058
*Ruta graveolens*	NC_045946	JQ230976
*Tetradium daniellii*	MZ145060	MN722092
*Tetradium ruticarpum*	MT134114	EU663534
*Toddalia asiatica*	MW194118	KM506903
*Zanthoxylum nitidum*	MN508801	MH558657

## APPENDIX C List of species and their GenBank accessions of published plastid markers obtained from GenBank in this study.

**Table:**

Species	GenBank accession		
	*matK*	*rpl16*	*trnL‐trnF*
*Boenninghausenia albiflora*	EF489070	EF489144	EF489218
*Chloroxylon swietenia / faho*	KX426057		AY295276
*Cneoridium dumosum*	EF489108	EF489182	EF489256
*Dictamnus albus*	EF489110	EF489184	EF489258
*Haplophyllum acutifolium*	EF489092	EF489166	EF489240
*Haplophyllum bastetanum*	EF489097	EF489171	EF489245
*Haplophyllum buxbaumii*	EF489095	EF489169	EF489243
*Haplophyllum dasygynum*	HM163990	HM163890	HM163790
*Haplophyllum dauricum*	EF489099	EF489173	EF489247
*Haplophyllum erythraeum*	EF489080	EF489154	EF489228
*Haplophyllum furfuraceum*	EF489093	EF489167	EF489241
*Haplophyllum latifolium*	EF489094	EF489168	EF489242
*Haplophyllum obtusifolium*	EF489098	EF489172	EF489246
*Haplophyllum villosum*	EF489096	EF489170	EF489244
*Psilopeganum sinense*	LT558092	LT558093	LT558094
*Ruta angustifolia*	EF489061	EF489135	EF489209
*Ruta chalepensis*	EF489049	EF489123	EF489197
*Ruta corsica*	EF489054	EF489128	EF489202
*Ruta graveolens*	EF489057	EF489131	EF489205
*Ruta microcarpa*	EF489068	EF489142	EF489216
*Ruta montana*	EF489063	EF489137	EF489211
*Ruta oreojasme*	EF489069	EF489143	EF489217
*Ruta pinnata*	EF489066	EF489140	EF489214
*Thamnosma hirschii*	EF489071	EF489145	EF489219
*Thamnosma montana*	EF489072	EF489146	EF489220
*Thamnosma pailensis*	EF489074	EF489148	EF489222
*Thamnosma socotrana*	EF489073	EF489147	EF489221
*Thamnosma texana*	EF489075	EF489149	EF489223
